# Gaming addiction among Tunisian adolescent

**DOI:** 10.1192/j.eurpsy.2021.2178

**Published:** 2021-08-13

**Authors:** S. Omri, M. Daoud, N. Smaoui, R. Feki, N. Charfi, J. Ben Thabet, L. Zouari, M. Maalej Bouali, M. Maalej

**Affiliations:** 1 Department Of Psychiatry “c”, Universty Hospital of Hedi Chaker, sfax, Tunisia; 2 Psychiatry C Department, Hedi chaker University hospital, sfax, Tunisia

**Keywords:** gaming, Addiction, adolescent

## Abstract

**Introduction:**

Gaming is a source of addiction for adolescents. It is recognized as a behavioral and mental health condition, both by the American Psychiatric Association and by the World Health Organization.

**Objectives:**

To determine the prevalence of gaming addiction among secondary school students.

**Methods:**

This cross-sectional study was conducted between September and October of 2020 among students enrolled in secondary school. The participants had filled the Game addiction scale and a data file regarding the socio-demographic information, physical and information about the internet access and use.

**Results:**

The initial sample was composed of 180 secondary school students. Among them 28 were excluded because they did not play video games. Final sample consisted of 152 students (90 males, 62 females) with a mean age of 13.14 ± 1.2 years. The average duration of connection among participants was 5.3 hours per day. Nearly one quarter of the participants (24,3%) played videogames more than 20 h per week. The prevalence of gaming addiction was 21,7%. The participants with gaming addiction were, on average, younger than those who were not addicted to gaming Game-addicted individuals were more likely to be male than female (13,8% vs 7,9%; p=0,036). There was, also, a significant relation between IA and having academic difficulties (p=0.042).

**Conclusions:**

Based on our study findings, that gaming addiction is a challenging problem among Tunisian adolescents. We recommend authorities consider gaming addiction a serious problem for the young population and make this growing phenomenon an adolescent health priority.

**Disclosure:**

No significant relationships.

